# Implementation, uptake and use of a digital COVID-19 symptom tracker in English care homes in the coronavirus pandemic: a mixed-methods, multi-locality case study

**DOI:** 10.1186/s43058-022-00387-y

**Published:** 2023-01-17

**Authors:** Pauline A. Nelson, Fay Bradley, Akbar Ullah, Will Whittaker, Lisa Brunton, Vid Calovski, Annemarie Money, Dawn Dowding, Nicky Cullum, Paul Wilson

**Affiliations:** 1 Division of Nursing, Midwifery & Social Work, School of Health Sciences, Faculty of Biology Medicine and Health, The University of Manchester, Room 6.312, Jean McFarlane Building, Oxford Road, Manchester, M13 9PL UK; 2 Manchester Centre for Health Economics, Faculty of Biology Medicine and Health, The University of Manchester, Jean McFarlane Building, Oxford Road, Manchester, M13 9PL UK; 3 Division of Population Health, Health Services Research & Primary Care, School of Health Sciences, Faculty of Biology Medicine and Health, The University of Manchester, Williamson Building, Oxford Road, Manchester, M13 9PL UK

**Keywords:** Care homes, Social care, Digital interventions, Implementation, Mixed-methods

## Abstract

**Background:**

COVID-19 spread rapidly in UK care homes for older people in the early pandemic. National infection control recommendations included remote resident assessment. A region in North-West England introduced a digital COVID-19 symptom tracker for homes to identify early signs of resident deterioration to facilitate care responses. We examined the implementation, uptake and use of the tracker in care homes across four geographical case study localities in the first year of the pandemic.

**Methods:**

This was a rapid, mixed-methods, multi-locality case study. Tracker uptake was calculated using the number of care homes taking up the tracker as a proportion of the total number of care homes in a locality. Mean tracker use was summarised at locality level and compared. Semi-structured interviews were conducted with professionals involved in tracker implementation and used to explore implementation factors across localities. Template Analysis with the Consolidated Framework for Implementation Research (CFIR) guided the interpretation of qualitative data.

**Results:**

Uptake varied across the four case study localities ranging between 13.8 and 77.8%.

Tracker use decreased in all localities over time at different rates, with average use ranging between 18 and 58%. The implementation context differed between localities and the process of implementation deviated over time from the initially planned strategy, for stakeholder engagement and care homes’ training. Four interpretative themes reflected the most influential factors appearing to affect tracker uptake and use: (1) the process of implementation, (2) implementation readiness, (3) clarity of purpose/perceived value and (4) relative priority in the context of wider system pressures.

**Conclusions:**

Our study findings resonate with the digital solutions evidence base prior to the COVID-19 pandemic, suggesting three key factors that can inform future development and implementation of rapid digital responses in care home settings even in times of crisis: an incremental approach to implementation with testing of organisational readiness and attention to implementation climate, particularly the innovation’s fit with local contexts (i.e. systems, infrastructure, work processes and practices); involvement of end-users in innovation design and development; and enabling users’ easy access to sustained, high-quality, appropriate training and support to enable staff to adapt to digital solutions.

**Supplementary Information:**

The online version contains supplementary material available at 10.1186/s43058-022-00387-y.

Contributions to the literature •Implementation framework-informed digital innovation in care homes is rare. This rapid evaluation applies such principles to examine uptake, use and implementation of digital innovation in care homes in response to COVID-19.

•We identified implementation factors influencing variation in initial uptake and use of the innovation in four case study localities, as well as decline in use over time across all localities, highlighting an interaction between internal and external contexts.

•We draw on the pre-pandemic e-health digital solutions evidence base to propose key factors to inform future implementation of rapid digital responses in care home settings even in times of crisis.

## Background

### The COVID-19 pandemic and care homes in England

In the early stages of the COVID-19 pandemic in the UK, the NHS was prioritised over social care, leading to the rapid discharge of approximately 25,000 untested people into care homes for older people between 17 March and 15 April 2020, contributing to the spread of infection, exacerbated by staff working across homes [1, 2]. Between March 2020 and April 2021, 41,675 care home residents died of COVID-19, over a quarter of all COVID deaths in England over the same period [3]. The pandemic added to the demands upon a social care sector already pressurised by funding and staffing issues [1, 2, 4, 5].

UK government and medical bodies recommended a range of infection control measures for care homes, including the use of assessment tools to monitor residents’ COVID symptoms/early deterioration, log care plans and ensure that general practitioners (GPs) could monitor/advise on residents’ health [6, 7]. In May 2020, NHS England requested that GP practices adopt a series of measures to support care homes, including virtual weekly rounds for residents [8]. This was followed by a Primary Care Network (PCN) Directed Enhanced Service (DES) [9] that required all care homes in England to have a named GP implement a weekly ‘home round’ to discuss residents identified as requiring review.

#### Digital COVID symptom tracker

In response to the national recommendations [6, 7], Greater Manchester (GM) introduced a digital COVID symptom tracker, for frequent collection of residents’ COVID symptoms and other key indicators. The tracker was recommended by the NHS Innovation Accelerator programme [10] which aims to support the uptake and spread of proven, impactful innovations across the NHS in England.

The tracker was designed for care home staff use on existing care home PCs, laptops, tablet devices, and other mobile devices to optimise the capacity for bedside assessment of residents’ health status and identifying early signs of deterioration through completion of data fields (Additional file 1). This data would be available to care home staff and shared directly with the resident’s designated care team. Data were intended to be conveyed in real time to facilitate the targeting of system responses to residents with the greatest need.

#### Implementation strategy

An agency mandated to support the adoption and spread of innovation in the NHS was responsible for implementation. Agency staff worked with the tracker’s developers and clinical leads in one defined GM geographical area (Locality 1, the informal pilot), to design a strategy for rapid implementation of the tracker in that locality’s care homes, followed by a rapid, sequential roll-out to care homes in the nine remaining localities of GM between April 2020 and March 2021. The planned implementation strategy involved three components: stakeholder engagement at strategic/operational levels, implementation support and training of care home staff to use the tracker (Table 1).

**Table 1:** COVID-19 digital tracker: planned implementation strategy

Component	Planned activity	Channels
*Stakeholder engagement*	***Strategic***	
	• Strategic agreement of project at GM health and social care system level	GM health and social care governance and emergency decision-making groups
	• Detailed briefing explaining rationale for decision, what will happen and when, to be shared with key stakeholder groups: Directors of Adult Social Services (DASSs) and wider Local Authority staff; care home managers and staff; residents and carers; Clinical Commissioning Groups (CCG); GPs; NHS Trusts; regional health and social care partnership organisation; regional combined authority organisation; regional mayor; integrated care team directors; implementation agency staff; technology developer staff; regional communications team; public engagement groups; local/national media	Regional health and social care partnership organisation; regional communications division; Directors of Adult Social Care (DASSs) for GM
	• Clinical Reference Group (CRG) to be established to oversee clinical aspects of tracker and its future development (to include representation from: GPs, NHS Trusts, DASSs and other Local Authority staff, care home managers/staff, clinical commissioning group staff, regional health and social care partnership organisation staff, technology developer staff)	Implementation agency staff
	***Operational***	
	• Locality steering groups to be established to ideally include for each locality: clinical lead, project lead, care home manager lead, information governance lead, GP representative, CCG contact, Local Authority contact	Implementation agency staff
	• Letter to care home managers to introduce ‘onboarding’ of care home	Local Authority or NHS locality lead with remit for adult social care homes
	• Welcome email to care homes with log-in instructions, user guide, frequently asked questions	Technology developers
	• Follow-up call for troubleshooting	Technology developers and/or implementation agency staff
	• Letter to GPs	Senior responsible officer
*Implementation support at the locality level*	• Support localities to access IT kit/wi-fi connectivity • Establish locality steering group for local deployment • Support training activity • Support clinical teams to integrate data and reporting from care homes • Identify and use GP and care home manager champions • Support localities with information governance arrangements	Implementation agency staff
*Training for care home staff*	• Training care homes to use IT equipment and tracker interface to complete twice-weekly assessments of residents’ COVID symptoms, confusion symptoms and general wellness • Based on developers’ prior experience of implementing similar technology in care home sector • 10–15 min, one-to-one, light-touch technical ‘on-boarding’ delivered via telephone by developer and/or implementation agency staff • Involved rapid familiarisation of care home staff with technical/functional aspects of tracker • Less focus on rationale for tracker and understanding tracker question fields • Supported by developers’ help pages • No or low-level follow-up	Implementation agency and/or technology developer staff

We were asked to conduct an independent evaluation of the implementation tied to uptake and use of the tracker, which were the main outcomes of interest to the regional health and social care system. A parallel study examining the impact of the tracker on COVID-19 spread is reported elsewhere [11].

### Implementation of digital interventions in care homes

Studies of digital/non-digital innovations in care homes have rarely used established implementation frameworks; however, this small literature emphasises the importance of co-production, training, compatibility with work processes and organisational readiness in these settings [12–17], highlighting in particular, concerns about poor existing digital infrastructure and capability [14, 17].

Our study examined the implementation, uptake and use of the digital COVID symptom tracker in regional care homes in the first year of the pandemic. Objectives were to (1) observe and record uptake and use across care home sites in different geographical localities and (2) explore processes of implementation across these localities and identify factors that may explain differences in uptake and use. As a rapid evaluation of the implementation/scale-up of a digital innovation aiming to mitigate adverse impacts on vulnerable populations, it addressed several of the criteria for responsive research on implementation in the time of COVID [18].

## Methods

### Study design

This rapid evaluation of implementation tied to outcomes took a multi-locality case study approach focusing on those localities where implementation was planned prior to December 2020, to enable use of the tracker to become established and sufficient usage data to be collected, to demonstrate trends over time (four case study localities). The study adopted a mixed-methods convergent parallel design, whereby quantitative and qualitative components were equally prioritised, carried out concurrently and analysed separately, and once analysis was completed, the results were integrated through team discussion during the overall interpretation of findings [19].

### Uptake and use of the tracker: data sample, duration and analysis

Tracker data were provided via regional COVID-19 dashboards. To derive the rate of uptake, GM care homes data were collated from the Care Quality Commission (CQC) care registry and merged with the tracker data. The rate of tracker uptake was calculated for each of the four case study localities using the number of care homes taking up the tracker (defined as a care home assessing and inputting residents’ data for at least one day between April 2020 and March 2021) as a proportion of the total number of care homes in a locality.

Use of the tracker was defined as the percentage of residents assessed [(assessed residents/total residents)×100]. Use at the four case study localities was then summarised by descriptive statistics (means). Mean use was also compared between 2020 and 2021 (to reflect pre- and post-vaccination roll-out) and weekday-vs-weekend at the locality level, and across all four localities. Analyses were performed in STATA 14.0.

### Evaluation of implementation factors

This component is reported according to SRQR [20] (Additional file 2).

#### Sampling, recruitment and data collection

Ethics approval was obtained prior to purposive and snowball sampling to generate a maximum variation sample of stakeholders to capture a wide range of perspectives on tracker implementation. Stakeholders at the four case study sites were sampled at four staff levels: (1) locality leads responsible for facilitating implementation into care homes in their area, (2) staff of care homes using the tracker, (3) clinicians responsible for the care of home residents and (4) implementation leads responsible for supporting regional tracker adoption and spread. Potential participants were identified via the implementation agency and by word-of-mouth. As far as possible, the care homes from which staff were recruited were also sampled purposively on varying characteristics to obtain a mix of homes; convenience sampling was adopted for some localities with fewer participating homes.

Data were collected via in-depth, semi-structured interviews (conducted by PAN, FB, LB and AM), with a topic guide broadly informed by the implementation literature [21, 22] to explore barriers, challenges and enablers involved in tracker implementation and how it impacted on care processes/practices of care home staff and clinicians (Additional file 3). All interviews were virtual (by telephone or video), August 2020–March 2021; participants gave informed consent before data collection.

#### Data analysis

Interviews were audio-recorded, transcribed and exported to NVivo 12 Pro software for data coding and management [23]. The Consolidated Framework for Implementation Research (CFIR) [21, 24, 25] was used with a Template Analysis (TeA) approach to guide thematic analysis of interview data [26, 27], enabling comparison/contrast of participant perspectives from different organisational contexts. Analysis involved familiarisation with interview transcripts; preliminary labelling of early data using the five CFIR domains and their constructs as an inclusive, deductive coding template, noting to what extent CFIR constructs accounted for the data gathered; coding of further data to modify the template (in this case, reducing the template by removing CFIR constructs judged as redundant); clustering of codes according to the most salient CFIR constructs to produce a final template; application of the final template to the full dataset; and drawing together key, interpretative, cross-cutting themes that captured the richest and most detailed aspects of the data [27]. The research team met regularly to discuss key insights from the analysis, closing data collection when analysis was judged to be theoretically sufficient.

## Results

Care homes’ uptake and use of the tracker in the four localities is presented followed by an analysis of implementation factors across the localities.

### Care homes’ uptake of the tracker across localities

During the planned implementation period (April 2020–March 2021), 144 GM care homes took up the tracker (24.9%) and uptake was recorded in eight of the 10 GM localities. Within the four case study localities, 91 care homes (44.2%) took up the tracker during this period.

Uptake varied across the four case study localities (see Table 2). There were similar levels of high uptake in the first two localities to implement the tracker (Locality 1, 77.8%; Locality 2, 76.7%). Uptake was lowest in Locality 3 (13.8%), followed by Locality 4 (15.9%).

**Table 2:** Care home tracker uptake in the four case study localities

	Locality 1	Locality 2	Locality 3	Locality 4	All four localities
Total no. of care homes	54	43	65	44	206
No. of care homes taking up^a^ tracker	42	33	9	7	91
Uptake (%)	77.8	76.7	13.8	15.9	44.2
First care home start date	Apr 20	Aug 20	Oct 20	Nov 20	N/A

^a^Uptake is defined as a care home assessing and inputting residents’ data for at least 1 day between April 2020 and March 2021

### Care homes’ use of the tracker across localities

**Fig. 1:**
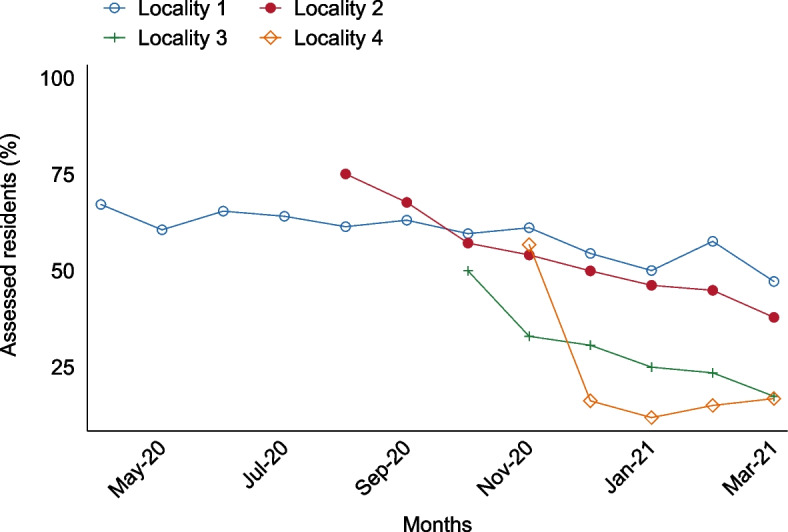
Usage trends across case study localities

Figure 1 plots average use of the tracker ([assessed residents/total residents]×100), by month, across the four case study localities over the period April 2020–March 2021. Use decreased in homes in all localities over time, at different rates. Over the sample period, average use ranged between 18 and 58% across localities: 58% in Locality 1; 50% in Locality 2; 25% in Locality 3; and 18% in Locality 4 (Table 3). Average use declined in 2021 (post-COVID vaccination roll-out) for all localities compared with 2020. Use was higher during the week than at weekends for all localities.

**Table 3:** Tracker use (use =[assessed residents/total residents]×100) across case study localities by year and day of week

	Locality 1	Locality 2	Locality 3	Locality 4	All four localities
First care home start date	April 2020	August 2020	October 2020	November 2020	
Average use^a^	58.07%	50.26%	24.54%	18.02%	52.72%
Average use 2020^b^	60.93%	56.67%	31.61%	26.60%	58.66%
Average use 2021^c^	50.48%	42.88%	21.60%	14.46%	42.37%
Average use over weekdays	68.91%	56.99%	31.36%	24.59%	62.00%
Average use over weekends	30.46%	33.04%	7.05%	0.00%	28.93%

^a^Data on use was assessed over the period April 2020 to March 2021

^b^2020 use covers the period April 2020 to December 2020

^c^2021 use covers January 2021 to March 2021

### Interview sample

We conducted 51 interviews across the four localities, including 24 staff (mainly managers) from 23 care home sites (Additional file 4). Participating care homes were balanced on case-mix (i.e. residential/nursing); however, the majority were medium/large, with CQC rating ‘good’, and with 20–59 staff (Additional file 5). The mean duration of interviews was 44 min.

### Implementation factors influencing uptake and use of the COVID-19 symptom tracker

**Fig. 2:**
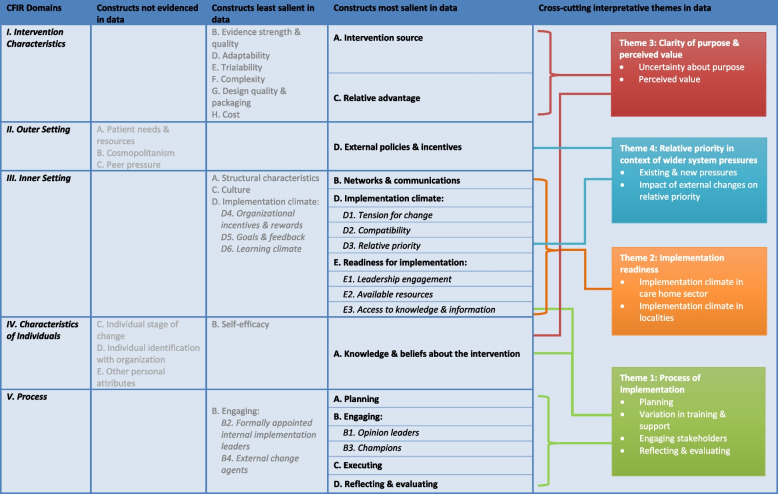
CFIR constructs: salience in qualitative data and final interpretative themes

Informed by CFIR constructs [28], we generated four interpretative themes reflecting the most influential factors that appeared to affect uptake/use of the tracker: (1) the process of implementation, (2) implementation readiness, (3) clarity of purpose/perceived value and (4) relative priority in the context of wider system pressures. Figure 2 displays the CFIR constructs found to be most salient in the interview data and which formed the basis for the final cross-cutting interpretative themes; least salient constructs and constructs that were not evidenced in the data are also displayed.

Supporting quotations for each theme from care home staff, clinician, locality and implementation lead participants are presented in Additional file 6.

### Theme 1: the process of implementation

The process of implementation deviated over time from the planned strategy (Table 1), for stakeholder engagement and care homes’ training; the implementation context also differed between the four case study localities (Table 4). Deviations and contextual differences are highlighted throughout this section.

**Table 4:** Description of implementation context in the four case study localities

GM Localities	Start date and number of care homes using tracker (at time of interviews)	Existing infrastructure, work processes and stakeholders involved	Training received by care home interview participants
***Locality 1***	Apr 2020 Majority of 54 homes across locality; roll-out mandatory.	**Clinicians:** Prior to roll-out all care homes already remotely supported by a digital hub of clinical staff. Hub backed by mature digital reporting systems, and lead clinicians closely involved in developing/piloting both the falls app and the COVID-19 symptom tracker in a small number of locality homes, and linking with a team of community pharmacists. **LA:** Involved in brokering of relationships with care homes and promotion of tool	Mixture of ‘light touch initial’ and'in-depth’ one-to-one model
***Locality 2***	Aug 2020 Majority of 43 homes across locality; roll-out advisory.	**Clinicians:** One GP practice covering residents in most homes co-located with palliative care/dementia nursing staff and pharmacist. Lead GP supportive in principle of tracker implementation. Digital infrastructure challenged. **LCO**: Existing remit for care home quality and brokered relationships with homes. **Health improvement organisation:** Project management of tracker roll-out; follow-up of homes post on-boarding and tracking data input.	‘In-depth’ group webinar model
***Locality 3***	Oct 2020 9 of 65 homes across locality; roll-out voluntary.	**Clinicians:** No single GP practice covering care homes; some practices aligned with particular homes but generally different GP practices covered different residents. Low interest in tracker among GPs. **CCG:** Involved in some brokering of relationships with care homes and informal follow-up. **LA:** Mainly assisted in helping to provide digital kit to homes.	‘Light-touch subsequent’ model
***Locality 4***	Nov 2020 7 of 44 homes across locality; roll-out voluntary.	**Clinicians:** No single clinical team linking with homes but multiple GP practices covering different residents. Low level of engagement among GPs; digital infrastructure challenged. Team of community nurses/AHPs providing support to homes for COVID response but low engagement with tracker. **LA**: Involved in brokering relationships with homes and assisting with project set-up; providing support to homes; linking with CCG. **CCG**: Engaging PCNs to promote tracker; linking with LA.	‘Light-touch subsequent’ model

*CCG* Clinical Commissioning Group, *LA* local authority, *LCO* local care organisation, *AHP* Allied Health Professional

#### Piloting

The tracker was implemented from April 2020 in Locality 1, where some care homes were already familiar with a similar digital tool and where local clinical stakeholders had helped develop the tracker for compatibility with local conditions. Rapid sequential roll-out across the remaining nine regional localities was planned. Further developmental work in other localities was constrained by the need for a rapid solution to address the unfolding crisis in care homes. A guiding assumption of the implementation team was that, following its adoption in Locality 1, the tracker’s advantages would be readily visible to other localities leading to straightforward uptake/use elsewhere; consequently, the tracker was implemented in Localities 2–4 without adjustment for differing local conditions.

#### Variation in training and support offered to care homes

Over the implementation period and across localities, care homes received differing levels of training and support to use the tracker. Additional file 7 shows the variation in training received by care homes whose staff were interviewed.

Training began in early adopter Locality 1 care homes, with a rapid ‘light-touch initial’ model, including advice to care home staff about assessing residents via the tracker approximately twice weekly. However, following queries about data completeness/quality from Locality 1 clinicians, a more intensive model, involving additional resources/follow-up, was delivered one-to-one to remaining Locality 1 homes and assessment guidance changed from twice weekly to daily before 11am. This more ‘in-depth’ approach was also used to train Locality 2 homes, though collectively via webinar. At this point, some implementers suggested the effort involved in delivering in-depth training to a sector where staff turnover was high could not be justified and that providing the intensive training model was taking too long in the urgency of the pandemic. By the time implementation commenced in Localities 3 and 4, training had reverted to a ‘light-touch subsequent’ version. Thus, training and support for care homes to use the tracker as well as expectations about resident assessments changed during the implementation period and may have affected staff’s understanding of the tracker’s purpose and how to use it (see Theme 3).

Privileging training speed over thoroughness not only impacted homes’ understanding but meant some missed out on training entirely if they could not fit in with implementers’ schedules. Additionally, implementers did not see the provision of ongoing support for tracker use as part of their role, meaning structures for helping homes maintain use were unaddressed. However, some implementers and locality leads stressed that fostering a learning climate where care home staff were supported to learn new skills was particularly important in a sector where access to training and skills development were relatively rare. The ‘lighter-touch’ training which came to be prioritised was felt to be at odds with this.

#### Engaging stakeholders

Although the implementation strategy (Table 1) included stakeholder engagement at strategic and operational levels, obtaining buy-in from strategic staff in the localities appeared to be prioritised over that of intended end-users of the tracker, i.e. care home staff and clinicians, particularly in Localities 2–4. Subsequent, intensive attempts to engage these stakeholders were made, but the lack of early involvement adversely affected their engagement with the tracker.

The importance of involving care home champions to help drive implementation across homes was also overlooked, and while highly engaged clinical champions from Locality 1 had co-designed the tracker for the local context, this model was transferred to other areas without accounting for differing conditions. In Locality 4 for example, implementers were unable to engage with the multiple GP practices attached to care homes, meaning that implementation happened largely without the involvement of GPs.

#### Reflecting and evaluating

Due to the rapid pace of implementation in pandemic circumstances, internal reflective learning among implementers was another element that was de-prioritised. With hindsight, some implementation leads questioned whether the hurried pace had been advisable, and others suggested that an organisational over-emphasis on the positive aspects of implementation neglected a focus on learning from drawbacks.

### Theme 2: implementation readiness

Readiness for tracker implementation was influenced by both the nature of work in the care home sector, and contextual differences between localities, making its roll-out more or less compatible with each area.

#### Implementation climate in the care home sector

While the tracker was intended for use by all levels of staff in care homes, home managers across localities largely sought to retain control of data input rather than delegating this task, suggesting a low readiness to spread its use among the workforce. Some managers did not wish to ‘burden’ staff with ‘extra’ tasks they deemed unsuitable for non-office-based roles, while others reported they wanted to ensure data input was complete and accurate. This retention of control by managers/office-based staff inhibited the diffusion of the tracker throughout the workforce and had the unintended effect of reducing data completeness, since data input was largely confined to weekdays when managers were on site. This may partially explain the ~30% reduction in use of the tracker during the weekends demonstrated earlier (Table 3).

#### Implementation climate in the localities

Existing health and social care structures/communication channels differed across Localities 1–4 (Table 4), meaning areas varied in their readiness for implementation.

Differences in locality buy-in and related attitudes to compliance monitoring meant pressure to adopt the tracker varied by area; this appears to be linked to the variation in tracker uptake seen in Table 2. In Locality 1 (the pilot), where there had been a degree of co-production and completion was monitored by the Local Authority, adoption was mandatory, meaning most homes took up the tracker. A majority of homes also took up the tracker in Locality 2 where adoption was ‘advisory’. In Localities 3 and 4, participation was voluntary, suggesting buy-in was less strong, with a small number of homes in each participating. Compliance was not formally monitored outside Locality 1.

The level of integration of health and social care in localities also appeared to affect the readiness for implementation of a tool aiming to link the social and primary care sectors. The systems in Localities 1 and 2 were said by implementers to be ‘quite well integrated’ compared with other areas, with good working relationships between organisations that strengthened implementation readiness. In both these localities, care homes had access to a single clinical team meaning support structures/lines of communication were more streamlined and beneficial to implementation. Locality 1 had the further advantage of being served by an existing digital hub for remote monitoring of care homes, a key facilitator for implementing a digital innovation.

In addition to differences in remote monitoring capabilities, some localities were more digitally enabled than others. For example, implementation support for equipment was not needed in Localities 1 and 2 (described as already ‘rich in kit’), while in Locality 3, the implementation team helped access equipment through a local charity. It was unclear whether any such support was provided to Locality 4, despite this area being described as digitally ‘immature’.

Overall, therefore, there was greater compatibility between the tracker and existing systems/processes in Locality 1, with implementers commenting that the innovation could not simply be dropped into other places where clinical support to homes was less integrated (i.e. where multiple GP practices served homes), and digital capability was lower.

### Theme 3: clarity of purpose and perceived value

Other implementation elements added to the uncertainty about the tracker’s purpose and in turn influenced views of its value among end-users.

#### Uncertainty about the tracker’s purpose

Care home staff were unsure how and from where the tracker had appeared and thus experienced its introduction as ‘top-down’. Viewing the tracker as an externally developed tool spoke to a lack of care home end-user involvement in its development and roll-out (Theme 1) and further added to the uncertainty around its purpose. The language used by some care home staff in relation to this (e.g. ‘I was under the impression…’, ‘I presume it is for…’, ‘from my point of view…’), suggested there had been a lack of clarity in fostering understanding of the tracker’s rationale. This uncertainty led staff to formulate their own beliefs about what the tracker was for, namely, collecting statistics to benefit other organisations regionally/nationally and not necessarily to help homes. Additionally, clinicians, locality leads and implementers all expressed doubts about whether care home staff understood the purpose of the tracker, with concern that this could affect accuracy of completion and compliance.

#### Perceived value of the tracker

While care home staff found the tracker technically straightforward and quick to use, some were concerned that question fields such as those on COVID symptoms were not sufficiently sensitive/specific to capture the nuances of health status in older people.

Overall, the value of the tracker was perceived more positively in Locality 1 and 2 care homes than those in Locality 3 and 4, but staff across localities had mixed views about the relative advantage of using the tracker. While there was no alternative solution for tracking residents’ COVID symptoms, the tracker was considered more useful for non-COVID aspects, such as storing information in one place, identifying missing ACPs and closer observation of residents’ general health using the Red-Amber-Green (RAG) rating. Indeed, care home managers expressed a willingness to work more digitally in the future with an adapted tracker for use beyond COVID.

Clinicians’ perceptions of the tracker’s relative advantage also differed between localities. In Locality 1, where the tracker had been designed, the value of the data generated by the tracker mirrored that of care homes in that it was also seen more positively by clinicians here than in other localities. For Locality 1 clinicians for example, the data were said to enable prioritisation of high-risk patients. Clinicians elsewhere were less positive, reporting that data generated by the tracker could be clinically limited and of less value. There were also indications that the views of care home staff and clinicians about the relative advantage of using the tracker mirrored each other, being particularly negative in Localities 3 and 4. In these localities, staff reported no change in communication and some questioned whether clinicians were looking at the data at all.

Outside Locality 1 therefore, tracker data was said not to have informed care decisions due to clinicians’ concerns about data completeness and quality. While a Locality 1 GP believed the tracker could enable PCNs to deliver requirements of the DES, GPs elsewhere did not see it replacing their existing care home processes. Indeed, within the period of the evaluation, no locality had formulated plans to make future funding of the tracker part of the contractual requirements for PCNs.

### Theme 4: relative priority in the context of wider system pressures

System pressures and the simultaneous introduction of national policies and incentives associated with the COVID-19 pandemic affected the degree to which care homes perceived a need for the tracker (tension for change) and the relative priority of its uptake/use.

#### Existing and new system pressures

Unprecedented additional pandemic-related work, such as managing COVID outbreaks, visitor policies/procedures and workforce shortages due to staff isolation, placed additional strain on an already pressurised care sector. Some implementers felt that the approach to implementation did not sufficiently recognise this. During times when homes were dealing with a COVID outbreak for example, managers were often so busy that completion of the tracker was de-prioritised. These pressures, alongside a lack of funding for staff development in the care sector, were felt to affect staff’s ability to engage with new initiatives/training.

#### Impact of external changes on relative priority of the tracker

Soon after implementation began, care homes in all localities were tasked with completing multiple data returns (national, regional and local), some of which involved similar requirements to the tracker (e.g. identification of residents suspected of having COVID-19). Far from taking work away, this duplication added to staff’s work burden and impacted on their willingness to engage with data input.

The introduction of COVID testing also affected implementation. By August 2020, when areas outside Locality 1 were beginning to use the tracker, routine care home resident testing was more commonplace, and swabbing could identify infections (including asymptomatic infections), quicker than symptom recording. This had a direct effect on the relative priority of the tracker, as it lost value as an early warning mechanism.

The introduction of testing was followed by the COVID vaccination drive. Vaccination of care home staff and residents was prioritised from late 2020, leading to a complete pause in implementation in Localities 3 and 4 where homes had recently joined and a decline in average use of the tracker in all areas (Fig. 1/Table 3). Implementers’ attempts to re-engage homes with the tracker after this hiatus were thwarted, as the tracker had become even lower priority for homes at this time. Clinicians, particularly in Localities 3 and 4, also faced difficulties engaging with the tracker at a time when they were heavily involved in delivering vaccines. Locality leads in these two areas underlined these clinical challenges, agreeing that outer setting pressures had significantly affected the implementation of the tracker in unintended ways.

## Discussion

This multi-locality case study, drawing on uptake/usage data and analysis of stakeholder perspectives informed by CFIR, offers evidence on the challenges of implementing a digital innovation to track COVID-19 in care homes during the pandemic and highlights key factors to guide future rapid responses.

### Summary of findings

In summary, the tracker was implemented as a potentially valuable component of the COVID response in care homes, at a time of great uncertainty and perceived need. However, a lack of adaptation to varying locality contexts, deviations from the planned implementation strategy (i.e. inconsistent stakeholder engagement particularly at operational level and variation in training models) and the introduction of multiple competing interventions alongside the tracker, considered to be higher value, brought a corresponding shift in views of its relative priority. The tracker came to be seen as a ‘blunt’ tool for generating meaningful COVID symptom data and low priority for the health and care system.

The study identifies implementation factors that help explain the variation in the outcomes of uptake/use *between* localities as well as the decline in use *across* localities over time. It also enables insights to be drawn in relation to several implementation outcomes as indicators of implementation success [29].

#### Variation in the outcomes of uptake/use between localities

The highest uptake/use of the tracker was in Locality 1 where a number of facilitating factors were present: (a) development of the tracker in partnership with highly engaged local clinicians to ensure compatibility with locality processes/structures and with some familiarity with prior digital technology; (b) mature remote monitoring capability and digital enablement; (c) integrated primary care/social services; (d) in-depth training and support for homes; and (e) implementation before COVID testing and vaccinations were routinised/prioritised in the wider system. These aspects mapped to more positive views of the tracker and a higher perceived relative advantage among care home staff and clinicians than in other areas. Locality 2’s uptake/use of the tracker was the second highest with some positive views, and where factors c, d, and e were present, but a and b were not. Localities 3 and 4 had the lowest use and most negative views, with none of the facilitating factors present.

#### Decline in use over time across localities

Use of the tracker was not exceptionally high in any one geographical area, with a pattern of decline in use over time across all areas that may be explained by common implementation factors. While at the outset the need for COVID outbreak management in a care sector at greater risk of severe resident illness and death was high, the following factors militated against optimal implementation in all areas: (1) the rapid pace of implementation in the context of an already pressurised care sector with low access to skills training faced with additional pandemic-related work pressures, leading to lost opportunities for testing and adjustment of the tracker across areas and associated reflective learning; (2) a lack of co-production with care homes, affecting clarity of purpose and general perceptions of the tracker’s relative advantage as an outbreak management tool; (3) the concurrent introduction of multiple other external interventions to control infection, producing a decline in tension for change over time and a corresponding drop in the relative priority of the tracker across all areas; and (4) the non-inclusion of the tracker, at least within the evaluation period, in plans for the primary care DES contract for care homes, precluding its embedding at PCN level in any area.

#### Implementation outcomes

The analysis also allows insights into seven of eight implementation outcomes as indicators of implementation success [29]. It suggests that *adoption* (uptake of the tracker), *acceptability* (the perception among stakeholders that the tracker was agreeable/satisfactory), *appropriateness* (the perceived fit, relevance, or compatibility of the tracker), *feasibility* (the extent to which the tracker was successfully used) and *fidelity* (the degree to which the tracker was implemented according to the implementation strategy) were all higher in localities where key facilitating factors were present to some degree compared to localities where these factors were less evident or entirely absent. Analysis also indicates that *penetration* (the integration/embeddedness of a practice within a service setting) *and sustainability* were affected by barriers 1–4 above, associated with implementation in the context of COVID pandemic pressures. *Cost* was not applicable as this was covered by an implementation team grant.

##### Comparison with existing literature

Descriptive developmental studies from outside the UK on digital solutions to manage COVID-19 in care homes [30–33] crucially omit implementation information; our study helps address this gap.

The benefits of digital innovations are often assumed [34]; however, implementing such technology is always ‘multi-level and complex’ [35] (p.10), with high failure rates [36]. Our findings resonate with three key factors found by Ross and colleagues to facilitate the successful implementation of e-health innovations pre-COVID [35]: (1) an incremental approach to implementation with testing of organisational readiness and attention to implementation climate, particularly the innovation’s fit with local contexts (i.e. systems, infrastructure, work processes and practices); (2) involvement of end-users in innovation design/development; and (3) enabling users’ easy access to high-quality, appropriate training. The limited implementation literature on digital innovation in care homes [12–15] and the wider grey literature on digital solutions in both health and social care settings [37–40] echo the importance of these facilitators. Drawing from this digital solutions evidence base, we suggest that the above-listed factors may be key to informing the future development/implementation of rapid responses in care home settings even in times of crisis.

In identifying these minimally important factors, our study also points to the importance of how the internal and external contexts in which a digital innovation is implemented might intersect to affect its relative priority and success, elements recognised in the digital implementation literature as under-researched [35].

In terms of internal context, even prior to COVID, care homes’ readiness for technological change was already challenged given the sector’s lack of digital maturity [14, 17, 39] and its organisational culture (norms, values and assumptions) [36], adversely affected by enduring staffing pressures [41], i.e. a poorly paid, undervalued workforce habitually viewed as unskilled, lacking in national accreditation and with limited opportunities for career progression [4]. These contextual factors contributed to an implementation climate already marked by internal ‘culture stress’, encompassing perceived stress, strain and role overload [42]. This speaks to a particular need in care homes for carefully planned/executed stakeholder engagement and user training with co-design [37, 38, 40], to foster a sense of ownership and harness motivation [43] and embed workforce development with digital skill enhancement [39]. By contrast, as a result of pressure in the system to act fast, tracker implementation lacked the involvement of care home staff as co-designers or champions [43, 44] and efforts to encourage users’ understanding of the tool’s rationale and benefits [14] fell away. While understandable given the context, this approach failed to support the needs of localities/homes where digital technologies are not routinely used [39].

In terms of external context, COVID itself and subsequent infection control management measures brought unprecedented additional strain on an already pressurised care sector [45, 46], affecting the relative priority of the tracker by eroding staff capacity for absorbing digital change and focusing energies on other ways of minimising outbreaks. Echoing this analysis, a rapid international narrative review of interventions to reduce the spread of COVID-19 in care homes suggests the effectiveness of symptom/temperature-based screening was limited, while universal resident/staff testing was crucial [47]. Indeed, a parallel study of the tracker’s impact found that adoption did not appear to influence rates of COVID-19 among residents [11]. Notably, tracker use was reported to fall when COVID outbreaks occurred, an opposite effect of that intended, bringing to mind prior calls to anticipate the potentially paradoxical consequences of implementing digital solutions [34]. Additionally, the tracker did not address a clear gap for GPs with the result that it failed to embed into primary care-based incentives/structures, known facilitators of successful implementation [35]. Thus, implementation approaches also failed to anticipate the impact of existing and new pandemic-related system pressures on the care home sector.

##### Strengths and limitations

Strengths of the study include its multi-locality, multi-site, multi-stakeholder, mixed-methods case study design in real time during COVID-19 pandemic combining uptake/usage and process data with theoretically informed data collection and analysis. The qualitative sample involved some convenience sampling of homes, and recruitment of care home staff and clinicians was challenging in the pandemic context. We nonetheless succeeded in gathering rich participant insights and the overall trend of usage data suggests the key findings may hold regardless of setting.

## Conclusions

This study supports prior calls for implementers to recognise the introduction of digital solutions as complex changes taking account of the characteristics of the innovation, where it is to be implemented, how it will be used and by whom [48]. Our study uses the CFIR to demonstrate the multi-level implementation factors affecting uptake and use of a digital innovation (the COVID-19 symptom tracker) in care homes in the first year of the pandemic. We identify factors that help explain both the variation in uptake/use between and the decline in use across localities, highlighting the characteristics of internal and external context, which coalesced to effect a shift in the perceived relative priority of the tracker from high to low over time. Our study also offers evidence to future implementers of digital interventions in care homes which even in times of crisis can inform a rapid response, highlighting the need for better intervention development and testing to ensure compatibility with existing infrastructure and work processes; co-production/engagement with stakeholders; and carefully planned/executed user training and support. Given the digital immaturity of some care homes, care home staff may need more time to adapt to digital solutions, with ongoing training and support.

## Supplementary Information

**Additional file 1.** Tracker fields for completion.

**Additional file 2.** Standards for Reporting Qualitative Research (SRQR).

**Additional file 3.** Interview topic guide.

**Additional file 4.** Participant characteristics.

**Additional file 5.** Characteristics of care homes whose staff were interviewed.

**Additional file 6.** Qualitative data extracts.

**Additional file 7.** Variation in training models/components received by care home interview participants by locality.

## Data Availability

The data that support the quantitative findings of this study are available from the Greater Manchester Health and Social Care Partnership, but restrictions apply. The data are not publicly available and were used under licence for the current study. Data are, however, available from the authors upon reasonable request and with permission of the Greater Manchester Health and Social Care Partnership. The qualitative dataset generated and analysed during this study is in the form of anonymised interview transcripts. Transcripts are not publicly available but are held on a University of Manchester secure server in line with study ethical approval. Transcripts are available from the corresponding author on reasonable request.

## Abbreviations

ACP: Advance Care Plan
CFIR: Consolidated Framework for Implementation Research
CQC: Care Quality Commission
DES: Directed Enhanced Service
EoL: End of Life
GM: Greater Manchester
GP: General Practitioner
NHS: National Health Service
PCN: Primary Care Network
RAG: Red-Amber-Green
SRQR: Standards for Reporting Qualitative Research
