# Supplementary material for: Implementation, uptake and use of a digital COVID-19 symptom tracker in English care homes in the coronavirus pandemic: a mixed-methods, multi-locality case study
Source: Implement Sci Commun. 2023 Jan 17;4:7. doi: 10.1186/s43058-022-00387-y (PMC9843982; doi:10.1186/s43058-022-00387-y)
Supplement: Supplementary file 1 — Additional file 1. Tracker fields for completion. [file 43058_2022_387_MOESM1_ESM.docx]

**Additional File 1. Tracker fields for completion**

| **Field** | **Definition** |
| --- | --- |
| ***Regular completion: symptom data*** | |
| COVID symptoms | Temperature of ≥37.8° and/or a new dry cough |
| Confusion symptoms | Increased confusion in the last 24 hours |
| General wellness | Wellness over the preceding four weeks via a RAG (red-amber-green) rating:   - blue – not unwell - green – was unwell but now better - amber – was unwell and is getting better - red – not getting better or may be dying |
| ***Completion once: end-of-life (EoL) data*** | |
| Advanced Care Plan (ACP) | Yes/no |
| Do Not Attempt Resuscitation Order (DNAR) | Yes/no |
| Preferred place of death | Yes/no and location if applicable |
| Prescription for EoL medications | Yes/no |
