# Supplementary material for: Implementation, uptake and use of a digital COVID-19 symptom tracker in English care homes in the coronavirus pandemic: a mixed-methods, multi-locality case study
Source: Implement Sci Commun. 2023 Jan 17;4:7. doi: 10.1186/s43058-022-00387-y (PMC9843982; doi:10.1186/s43058-022-00387-y)
Supplement: Supplementary file 2 — Additional file 2. Standards for Reporting Qualitative Research (SRQR). [file 43058_2022_387_MOESM2_ESM.docx]

**Additional File 2. Standards for Reporting Qualitative Research (SRQR)**

| **No.** | **Topic** | **Item** |
| --- | --- | --- |
|  | ***Title and abstract*** |  |
| S1 | Title | Title concisely describes nature and topic of study, identifying mixed-methods, multi-locality case study approach. |
| S2 | Abstract | Abstract summarises key study elements using required journal format: background, method, results, conclusion. |
|  | ***Introduction*** |  |
| S3 | Problem formulation | Significance of problem described (how COVID outbreaks in care homes were managed with a new digital tool in early pandemic), with reference to prior empirical work including implementation science-informed care homes research. |
| S4 | Purpose or research question | Purpose of study and specific objectives identified (i.e., to examine uptake, use and implementation factors associated with introduction of a digital COVID symptom tracker in English care homes) in the early pandemic. Objectives were to: 1) observe and record uptake and use of the tracker across four case study localities where the tracker was implemented (in multiple care home sites); and 2) examine and explain any differences in uptake/use with reference to the implementation factors across geographical localities. |
|  | ***Methods*** |  |
| S5 | Qualitative approach and research paradigm | Mixed-methods case study approach informed by the Consolidated Framework for Implementation Research (CFIR). |
| S6 | Researcher characteristics and reflexivity | Data collection was conducted by experienced qualitative researchers who had no prior relationship with research participants. |
| S7 | Context | The context was a multi-locality, multi-site (care homes in a region of England) comparison of the implementation of a new digital tool for COVID management in the early part of the pandemic (use and process). |
| S8 | Sampling strategy | Qualitative sampling was conducted at several levels for maximum variation: by geographical locality, by type of home (large/medium/small; CQC rating; case-mix; staffing level) and by professional stakeholder role (care home staff, locality leads, implementation leads, clinicians). Data collection was closed when analysis was judged to be theoretically sufficient using CFIR constructs as a lens. |
| S9 | Ethical issues pertaining to human subjects | Ethical permission was granted (organisation and reference numbers included in the manuscript’s declarations) before the study began and informed, audio-recorded verbal consent was also obtained from all interview participants prior to data collection. Short illustrative quotations from research interviews included in the manuscript are anonymised. |

**Additional File 2. Standards for Reporting Qualitative Research (SRQR) (continued)**

| **No.** | **Topic** | **Item** |
| --- | --- | --- |
|  | ***Methods*** |  |
| S10 | Data collection methods | Data collected in one-to-one in-depth interviews; start/stop dates and mean length of interviews included in manuscript; qualitative process data triangulated with quantitative uptake/usage data. |
| S11 | Data collection instruments and technologies | Interviews semi-structured and based on a topic guide informed by implementation science literature (included as an additional file). Interviews conducted remotely in pandemic circumstances and audio-recorded. |
| S12 | Units of study | Analysis of implementation factors is by geographical locality to reflect the implementation approach. In addition to number and description of the implementation context in four case study localities, number and characteristics of participating care homes and professional stakeholder participants within participating localities are described. |
| S13 | Data processing | Interview audio files transcribed and anonymised, exported to NVivo 12 and coded. |
| S14 | Data analysis | Thematic analysis conducted using a Template Analysis approach with CFIR constructs as a lens to compare/contrast participant perspectives from different organisational contexts. Analysis involved: familiarisation with interview transcripts; preliminary labelling of early data using the five CFIR domains and their constructs as an inclusive, deductive coding template, noting to what extent CFIR constructs accounted for the data gathered; coding of further data to modify the template (in this case, reducing the template by removing CFIR constructs judged as redundant); clustering of codes according to the most salient CFIR constructs to produce a final template; application of the final template to the full dataset; drawing together key, interpretative, cross-cutting themes that captured the richest and most detailed aspects of the data. The research team met regularly to discuss key insights from the data, informed by CFIR constructs and closed data collection when analysis was judged to be theoretically sufficient. |
| S15 | Techniques to enhance trustworthiness | Sampling strategy clearly described/justified in the manuscript. Mixed-methods approach to data collection and analysis enabled triangulation of findings (quantitative uptake/usage data and qualitative stakeholder perspectives). The qualitative sample included multi-site and multi-locality professional perspectives from different levels to provide analytic depth and comprehensiveness. We used systematic analytic processes (Template Analysis informed by CFIR, a recognised, internationally used implementation framework) to code/analyse qualitative data. The analysis process was iterative and conducted jointly by a team of experienced researchers. |
| **No.** | **Topic** | **Item** |
|  | ***Results/findings*** |  |
| S16 | Synthesis and interpretation | Qualitative data synthesis informed by CFIR framework enabling key themes to be generated that appeared to be most influential in implementation efforts. Relationships between thematic areas drawn out. |
| S17 | Links to empirical data | Interpretation of qualitative data supported by detailed participant quotations and triangulated with quantitative usage data for analytic depth. |
|  | ***Discussion*** |  |
| S18 | Integration with prior work, implications,  transferability, and contribution(s) to the field | Main findings summarised, using qualitative synthesis of key implementation factors to explain variation in use between localities and trends over time across localities. How findings  and conclusions support and add to the prior implementation literature are presented, identifying novel contribution to field and actionable findings to inform further implementation programmes. |
| S19 | Limitations | Limitations included, i.e., sampling challenges and transferability. However, strengths also documented i.e., multi-locality, multi-site, mixed methods approach with common trends/themes that suggest the analyses may hold across localities. |
|  | ***Other*** |  |
| S20 | Conflicts of interest | Authors’ conflicts of interests listed (i.e., none). |
| S21 | Funding | Funding and disclaimer included in manuscript. |
