# Supplementary material for: Implementation, uptake and use of a digital COVID-19 symptom tracker in English care homes in the coronavirus pandemic: a mixed-methods, multi-locality case study
Source: Implement Sci Commun. 2023 Jan 17;4:7. doi: 10.1186/s43058-022-00387-y (PMC9843982; doi:10.1186/s43058-022-00387-y)
Supplement: Supplementary file 3 — Additional file 3. Interview topic guide. [file 43058_2022_387_MOESM3_ESM.docx]

**Additional File 3. Interview topic guide**

| **Topic** | **Prompts** | **Topic** | **Prompts** |
| --- | --- | --- | --- |
| *Role*  Professional background/current role  Role in implementing/using tracker |  | *Engagement*  Rationale for tool  How promoted?  Staff willingness to engage  Views of training/support provided  Extent of reach/spread |  |
| *Use*  How tool used?  Information gathered  Perceived fit with work practices | Data input by whom/how often? | *Perceived impact*  Work practices  Resident management/care decisions  Realised benefits | Impact on workload, workflow, referrals, communication with clinicians |
| *Expected value*  Perceived aim  Expected benefits/challenges |  | *Perceptions of implementation*  What went well and why?  Intended/unintended risks/challenges  Future development/improvements  Sustainability | How challenges overcome?  Barriers to/facilitators of sustainability |
| *Context*  Wider health and social care system  Care homes setting  Primary care setting | Networks and communications,  work processes and practices |  |  |
