# Supplementary material for: Implementation, uptake and use of a digital COVID-19 symptom tracker in English care homes in the coronavirus pandemic: a mixed-methods, multi-locality case study
Source: Implement Sci Commun. 2023 Jan 17;4:7. doi: 10.1186/s43058-022-00387-y (PMC9843982; doi:10.1186/s43058-022-00387-y)
Supplement: Supplementary file 4 — Additional file 4. Participant characteristics. [file 43058_2022_387_MOESM4_ESM.docx]

**Additional File 4. Participant characteristics**

| **Participant type** | **Locality (number of participants)** |
| --- | --- |
| Care Home Staff | Locality 1 (8)  Locality 2 (9)  Locality 3 (3)  Locality 4 (4)  **(24)** |
| Clinicians | Locality 1 (4)  Locality 2 (3)  Locality 3 (2)  Locality 4 (1)  **(10)** |
| Locality Leads | Locality 1 (2)  Locality 2 (3)  Locality 3 (2)  Locality 4 (2)  **(9)** |
| Implementation Leads | **(8)** |
|  |  |
| **GRAND TOTAL** | **(51)** |
