# Supplementary material for: Implementation, uptake and use of a digital COVID-19 symptom tracker in English care homes in the coronavirus pandemic: a mixed-methods, multi-locality case study
Source: Implement Sci Commun. 2023 Jan 17;4:7. doi: 10.1186/s43058-022-00387-y (PMC9843982; doi:10.1186/s43058-022-00387-y)
Supplement: Supplementary file 5 — Additional file 5. Characteristics of care homes whose staff were interviewed. [file 43058_2022_387_MOESM5_ESM.docx]

**Additional File 5. Characteristics of care homes whose staff were interviewed**

| **Locality** | **No. of homes** | **Size of home*** | **CQC rating** | **Staff levels** | **Case-mix** |
| --- | --- | --- | --- | --- | --- |
| Locality 1 | 7 | Small = 1  Medium = 3  Large = 3 | Outstanding = 2  Good = 5  RI = 0 | < 19 = 0  20-39 = 3  40-59 = 4  60-99 = 0  100+ = 0 | Residential only = 5  Nursing = 2 |
| Locality 2 | 9 | Small = 0  Medium = 4  Large = 5 | Outstanding = 0  Good = 8  RI = 1 | < 19 = 0  20-39 = 3  40-59 = 3  60-99 = 2  100+ = 1 | Residential only = 2  Nursing = 7 |
| Locality 3 | 3 | Small = 1  Medium = 1  Large = 1 | Outstanding = 0  Good = 3  RI^†^ = 0 | < 19 = 1  20-39 = 1  40-59 = 0  60-99 = 1  100+ = 0 | Residential only = 2  Nursing = 1 |
| Locality 4 | 4 | Small = 1  Medium = 2  Large = 1 | Outstanding = 0  Good = 3  RI = 0  Not inspected = 1 | < 19 = 0  20-39 = 3  40-59 = 0  60-99 = 0  100+ = 1 | Residential only = 3  Nursing = 1 |
| **Total** | **23** | **Small = 3**  **Medium = 10**  **Large = 10** | **Outstanding = 2**  **Good = 19**  **RI = 1**  **Not inspected = 1** | **< 19 = 1**  **20-39 = 10**  **40-59 = 7**  **60-99 = 3**  **100+ = 2** | **Residential only = 12**  **Nursing = 11** |

*Small [0–23 beds], Medium [24–40 beds], Large [41+ beds] (after Morciano and colleagues[30])

^†^ RI = Requires Improvement
