# Supplementary material for: Implementation, uptake and use of a digital COVID-19 symptom tracker in English care homes in the coronavirus pandemic: a mixed-methods, multi-locality case study
Source: Implement Sci Commun. 2023 Jan 17;4:7. doi: 10.1186/s43058-022-00387-y (PMC9843982; doi:10.1186/s43058-022-00387-y)
Supplement: Supplementary file 6 — Additional file 6. Qualitative data extracts. [file 43058_2022_387_MOESM6_ESM.docx]

**Additional File 6. Qualitative data extracts**

| **Themes/sub-themes** | **Quotations** |
| --- | --- |
| **Theme 1. The process of implementation** |  |
| ***Piloting*** |  |
| Rapid planning in pandemic context | *We’ve had to run at this gate like a bull and say, what can we get done and quickly? And… that’s not ideal, but it’s a reality that we’ve had to live with. So I think we’ve worked on a ‘good enough’ and fuelling the jet plane as the plane is flying kind of approach.* (IL4) |
| Expected smooth transfer from one locality to another | *[Other areas outside Locality 1] will see it working and understand it’s working.* (IL3) |
| ***Variation in training and support offered to homes*** |  |
| Difficulty using tracker with ‘light-touch’ training | *We’ve got homes that don’t really understand the questions and aren’t filling it in on a regular basis, and we need to bring some standardisation… it’s not just a case of telling people how to do something, they really need the understanding of why and the background.* (IL2) |
| Expectations for resident assessments not fixed | *It wasn’t really mandated…when [care homes] should be putting it in.* (IL2) |
| Intensive training too lengthy | *We’ve got to get that balance between… how do we get people on it properly, but how do we get people on it quickly?... at the moment I think the concerns around speed are winning over the concerns of data quality*. (IL5) |
| Differing levels of training and support to care homes | *They’ve not all had exposure to somebody explaining this is what it is, this is why it’s been developed, this is what it’s being used for, this is why we’re asking you to do it, these will be the benefits of it… that kind of educational stuff, and I think that’s probably why we’ve got some inconsistencies and people, you know, not understanding what it’s really for and what it’s meant for.* (IL2) |
| Maintenance structures neglected | *There’s been a slight disconnect in thinking that we [implementers] are doing all this for [the localities], but actually, no – it’s yours… what we’ve done is try to get everybody around the table in our [site] steering groups and then facilitate those conversations, rather than saying, ‘it’s the CCG’s responsibility, you’re leading on this for your locality’.* (IL6) |
| ‘Light-touch’ training inadequate for care home workforce | *It's not [care homes’] problem, it's our problem because we haven't instilled that understanding to them, because the care home sector are a very different sector with a different knowledge and skill-base, and I think… we've let them down.* (LL8, Locality 4) |

**Definitions**: IL – Implementation Lead; LL – Locality Lead

**Additional File 6. Qualitative data extracts (continued)**

| **Themes/sub-themes** | **Quotations** |
| --- | --- |
| **Theme 1. The process of implementation** |  |
| ***Engaging stakeholders*** |  |
| Lack of engagement with care home staff and clinicians | *With the benefit of hindsight… a much more structured approach to… engagement would have benefited us more. So in those very early days it was sent straight out to directors of adult social services and quickly sort of done, so I think we're paying for the pace at which we moved in the beginning now, because we're having to close that loop a few more times… there doesn't seem to be much weight given to requests coming from carers or local GPs. It definitely seems to be driven more by the strategic.* (IL1) |
| Lack of clinician champions outside Locality 1 | *We've got [GP] in Locality 1, who's a brilliant clinical champion… but he's just one man that is very involved in it, so you only ever hear the exceedingly positive things [about the tracker]. There doesn't seem to be that balance sometimes of, well, actually what's it like on the ground outside of that [site]?* (IL1) |
| Lack of ownership among clinicians outside Locality 1 | *Primary care clinical directors thought it was being ‘done to’ rather than them being ‘involved with’, so they just kind of cut off… if this programme… had a different engagement plan and that engagement plan had involved primary care from the outset, and the [PCNs], then it would be in a different position.* (LL7, Locality 4) |
| ***Reflecting and evaluating*** |  |
| Lack of time for reflective learning | *Our approach in relentlessly pushing the deployment of [tracker] and not necessarily taking a temperature check of what the system is like, I struggle with.* (IL1) |
| Questioning if pace of implementation was justified | *There’s a lot we did on the hoof which… in hindsight… whilst we were in it, it felt like… we’re in a situation, we need to respond, we need to get going. Sort of, reflecting back, maybe things weren’t as urgent as we thought they were and maybe there were things… [and] processes we didn’t need to bypass.* (IL5) |
| Need for balanced view of implementation lessons | *We do need to celebrate [successes]… but we do need to be honest and transparent and talk about the issues… and not just go, ‘oh, yes, it’s fine, don’t worry about it…’ it’s not being negative you know, it’s actually just that reality check and sorting out issues when they need sorting out. So, I’m not sure that there’s been perhaps the openness.* (IL2) |

**Definitions**: IL – Implementation Lead; LL – Locality Lead

**Additional File 6. Qualitative data extracts (continued)**

| **Themes/sub-themes** | **Quotations** |
| --- | --- |
| **Theme 2. Readiness for implementation** |  |
| ***Implementation climate in the care home sector*** |  |
| Doubts about readiness of care home workforce | *I like to do it myself so I know that everything is right, that’s my only concern.* (Care Home Manager 4, Locality 1)  *Well basically, at the moment, the senior staff have enough to do… and also, by us doing it, the manager and I, we’ve got some kind of control over it, because things get forgotten*. (Care Home Administrator 1, Locality 2)  *It’s a very disenfranchised, minimum wage workforce… and obviously, the care home wants to deliver good quality data to support their residents. So, if the care home manager thinks that they’re best placed to do that, I understand why.* (IL6) |
| ***Implementation climate in the localities*** |  |
| Remote monitoring capability not ready outside Locality 1 | *[Other localities] don’t have [a digital hub], do they? So I don’t know how realistically [the tracker] is going to work for them. I mean, I don’t know who’s going to be there to support them*. (Care Home Manager 4, Locality 1) |
| Differences in digital enablement between areas | *A lot of the homes didn’t have any devices that video calls could happen on, so we had a couple of occasions where GPs wanted to do video calls with residents and they were dependent on one of the care staff using their iPhone.* (LL6, Locality 3) |
| Maturity of locality systems impacting on readiness | *The systems within the localities are all at varying degrees of maturity, and I think Locality 1 is possibly the worst one that we could have launched with because it's so mature that it's almost set an artificial precedent of, well, it can be done in Locality 1, it can be done anywhere. And nowhere else really has that same mature relationship between the different organisations or the maturity of their digital health team responses… and it sometimes feels like we're trying to… take a cookie cutter approach to it and it definitely isn't a case of one size fits all.* (IL1) |
| **Theme 3. Clarity of purpose and perceived value** |  |
| ***Uncertainty about the tracker’s purpose*** |  |
| ‘Top-down’ introduction of tracker | *[Tool] was promoted in the Tuesday afternoon Zoom [care home meeting]. Yeah. And we were thinking ‘what is this tool? We’ve never seen this tool’.* (Care Home Deputy Manager 1, Locality 2) |

**Definitions**: IL – Implementation Lead; LL – Locality Lead

**Additional File 6. Qualitative data extracts (continued)**

| **Themes/sub-themes** | **Quotations** |
| --- | --- |
| **Theme 3. Clarity of purpose and perceived value** |  |
| ***Uncertainty about the tracker’s purpose*** |  |
| Beliefs about the tracker’s purpose among care homes | *I was trying to sort of work out who it was helping – the CCG, so they can keep an eye on what’s going on in the care homes, without having to actually contact us? I don’t know. (Care Home Manager 2, Locality 1)* |
| Doubts that care homes understood the purpose | *When we talked to [homes] when we were doing the follow up calls, we had a sense of, you know… did they get it? Like… somebody… asked me… the delirium question and she was saying, ‘why are they asking me about this person because she’s not got a urine infection?’ And you’re just like, yeah, you’ve missed the point on that one!* (IL5) |
| ***Perceived value of the tracker*** |  |
| Tracker relatively quick to complete | *It’s no bother, it doesn’t take any nursing minutes away* (Care Home Manager 8, Locality 2) |
| Tracker questions not sufficiently sensitive/specific | *It says, ‘have they got a temperature or a cough?’… and as we know… COVID-19… can be completely different in care settings… with this elderly population… it’s not sensitive enough… [residents] can develop a temperature just with a bit of dehydration or whatever… everybody’s sort of panicking and on high alert, you know? I don’t know if it needs to be a bit more specific that part… describing the symptoms?* (Care Home Manager 2, Locality 1) |
| Seeing value in an adapted tracker beyond COVID | *[It’s] not [helped] with COVID but I think it’s helped us with other infections, sort of urine infections, chest infections…that’s where I tend to find it’s quite useful. (*Care Home Manager 1, Locality 3)  *[Tracker is] very COVID related. It doesn’t highlight whether people have deteriorated from any other condition. So, if it’s to continue I think we need to add deterioration in other health conditions, you know, like… if someone’s got chest pains and he’s got a UTI, somebody’s got a chest infection, [tracker] doesn’t necessarily highlight it because… it’s aimed mainly at COVID.* (Care Home Manager 3, Locality 1) |
| Lack of response from clinicians in Localities 3 and 4 | *It just seems that we’re inputting data and I’m not 100 per cent sure actually where the data goes, if I’m honest, or what happens with it.* (Acting Manager 1, Locality 3)  *There’s no actual indicator that the data has been reviewed or seen by anybody... it’s almost like the one-way flow type situation.* (Care Home Manager 18, Locality 4) |

**Definitions**: IL – Implementation Lead; LL – Locality Lead

**Additional File 6. Qualitative data extracts (continued)**

| **Themes/sub-themes** | **Quotations** |
| --- | --- |
| **Theme 3. Clarity of purpose and perceived value** |  |
| ***Perceived value of the tracker*** |  |
| Value for clinically prioritising Locality 1 residents | *We were asked by our CCG to do proactive advanced care planning for all our high-risk patients in the care homes. So the tracker actually helped us to identify the cohort of the people.* (GP2, Locality 1) |
| Lack of clinical value of tracker data outside Locality 1 | *[There’s] not [much] information that I will say, well, I need to know that from the app…. I probably would look into having more meaty clinical things in it for me to appreciate it more.* (GP4, Locality 2) |
| Low value of tracker for care homes and clinicians in Localities 3 and 4 | *[Care home]… said, ‘well, it’s another layer of workload, it’s capacity’ and so that was one of the reasons why they’d pulled back [from using tracker] as well as me saying to them, ‘look, I don’t think it’s beneficial for us here’.* (GP7, Locality 3)  *Initially I did go on [the tracker dashboard] every day… when I realised that, unfortunately [some care homes] were not reporting regularly, it could be days and weeks. It just… didn’t really then have any value.* (AHP1, Locality 4) |
| **Theme 4. Relative priority in the context of wider system pressures** |  |
| ***Existing and new system pressures*** |  |
| Unprecedented additional pandemic-related work not taken into account | *I had one woman who nearly cried on me, because she’s just, like, ‘my god, I’ve got no deputy, my staff are off… everything else I’ve got to do for COVID, it’s nine hours a week!’ So that pressure they’re under, all the extra stuff they’ve got to do… they’ve got… all these other demands because of COVID on them.* (IL5)  *I still don’t think everybody in my organisation really gets what it’s like to be working in a care home… I don’t think they really have a true appreciation of what a very challenging environment it is irrespective of where we are now in the middle of a pandemic.* (IL2) |
| Tracker not completed during COVID outbreaks | *Even though it might only be a five minute job… it was just five minutes I didn’t have at the time.* (Care Home Manager 13, Locality 2) |
| Care home pressures precluding engagement with training | *They're such a stretched workforce that to get them to take some time out to come and do this… is very difficult, because the organisation isn't willing to pay for that time, so it had to be done in their own time.* (IL1) |

**Definitions**: IL – Implementation Lead; LL – Locality Lead

**Additional File 6. Qualitative data extracts (continued)**

| **Themes/sub-themes** | **Quotations** |
| --- | --- |
| **Theme 4. Relative priority in the context of wider system pressures** |  |
| ***Impact of external changes on relative priority of the tracker*** |  |
| Duplication of work for care home staff in using the tracker | *[Community health team], were phoning up every day asking all the same information as we were putting in the trackers… infection prevention were ringing, asking the same questions [as] the [national] Capacity Tracker [and] the COVID tracker. So… we’re having to give this information to so many different professionals, when you should all be linking up!* (Care Home Manager 2, Locality 1)  *The tracker needs to be really careful… about not duplicating things, because the [NHS] Capacity Tracker’s mandated centrally, so [homes] are going to have to do that one and they really... they [have] a lot of extra work compared to normal with having to swab test the staff and all of that and everything with COVID. We don’t want to just layer things on because it seems like a good idea.* (LL7, Locality 4) |
| Tracker losing value over time as an early warning mechanism | *We know quite quickly when an outbreak is happening… I’m not sure that the COVID-19 tracker on its own is really telling anyone that much at this point… now that we’ve got testing.* (LL7, Locality 4) |
| Vaccination programme impacting on implementation in homes | *But now even just being able to communicate with those homes at the highest level is very difficult because vaccinations have absolutely taken over everything… there’s so much going on in the system, because of that people have so little time, and so it can often create confusion because people aren’t having time to reflect on what work they’re doing.* (IL8) |
| Vaccination impacting on clinical capacity of GPs | *Capacity-wise and stuff, at the moment, just having to do the vaccines… I just don’t think that we’d thought about [the tracker] recently… it would probably actually save us time, but if we’re to be massively involved with the upheaval of planning it and getting it organised and getting it done, it’s not going to happen at the moment because we’re all basically double shifting with the vaccines… and we’re at 150 per cent our normal capacity.* (GP7, Locality 3) |

**Definitions**: IL – Implementation Lead; LL – Locality Lead

**Additional File 6. Qualitative data extracts (continued)**

| **Themes/sub-themes** | **Quotations** |
| --- | --- |
| **Theme 4. Relative priority in the context of wider system pressures** |  |
| ***Impact of external changes on relative priority*** |  |
| Prioritisation of other measures over the tracker by clinicians | *I don’t think, to be honest, it’s that [GPs] don’t want to engage, I just think that they are so busy. And especially now with all the vaccine roll-outs and everything else… I think now probably wouldn’t be the best time to push with it with our GPs.* (LL9, Locality 3)  *Well, the intention was a quick roll-out. But we had a lot of barriers on the clinical side. So – paused. And also paused in truth because pre-Christmas, there was all stuff about prepping for visitors going into care homes, then we went into lockdown and there has been all the vaccination roll-out stuff. It’s a really difficult, really challenging time to mobilise a new piece of software.* (LL7, Locality 4) |
| Low priority of the tracker for system | *Even given… [the] COVID crisis situation... when you think about [it], has it really left the systems thinking, ‘oh my God, I can't do without it!’ the answer is – no, it hasn't left us thinking that.* (LL8, Locality 4) |

**Definitions**: IL – Implementation Lead; LL – Locality Lead
