# Supplementary material for: Implementation, uptake and use of a digital COVID-19 symptom tracker in English care homes in the coronavirus pandemic: a mixed-methods, multi-locality case study
Source: Implement Sci Commun. 2023 Jan 17;4:7. doi: 10.1186/s43058-022-00387-y (PMC9843982; doi:10.1186/s43058-022-00387-y)
Supplement: Supplementary file 7 — Additional file 7. Variation in training models/components received by care home interview participants by locality. [file 43058_2022_387_MOESM7_ESM.docx]

**Additional File 7. Variation in training models/components received by care home interview participants by locality**

| **Training and support delivered to care homes**  **(Models and components)** | **Locality 1**  From April 2020 | **Locality 2**  From Aug 2020 | **Locality 3**  From Oct 2020 | **Locality 4**  From Nov 2020 |
| --- | --- | --- | --- | --- |
| 1. ***LIGHT TOUCH INITIAL MODEL***   *Components*  Training care homes to use IT equipment and tracker interface to complete twice-weekly assessments of resident’s COVID symptoms, confusion symptoms and general wellness  Based on developers’ prior experience of implementing similar technology in care home sector  10-15 minute, one-to-one, light-touch technical ‘on-boarding’ delivered via telephone by developer and/or implementation agency staff  Involved rapid familiarisation of care home staff with technical/functional aspects of tracker  Less focus on rationale for tracker and understanding tracker question fields  Supported by developers’ help pages  No or low-level follow-up | ✓  Early adopter homes |  |  |  |
| 1. ***IN-DEPTH MODEL***   *Components*  Slower-paced ‘educational-style’ training  Model 1 components (i.e., IT equipment and tracker interface use) plus addressing rationale for/anticipated benefits of tool and interpretation of tracker question fields to complete daily resident assessments before 11am  Supported by purpose-made training manual and slide-set developed by implementers  Delivered jointly (implementers leading, developers supporting)  Structured one-to-one telephone follow-up | ✓  Later adopter homes  (one-to-one online; 3 telephone follow-ups by implementers) | ✓  Phase 1 and 2 homes (group-based webinar; 2 telephone follow-ups (1 by developers, 1 by implementers) |  |  |
| 1. ***LIGHT-TOUCH SUBSEQUENT MODEL***   *Components*  Rapid one-to-one ‘on-boarding’ by developers with emphasis on technical/functional aspects of using the tracker interface to complete daily resident assessments before 11am  Access to drop-in training webinars (open to any care home; any locality) run by implementers with tracker demonstrations by developers  Delivered jointly (developers leading, implementers supporting) or solely by developers  Homes referred to materials developed in 2 (in-depth model)  No or low-level follow-up |  |  | ✓  All homes | ✓  All homes |
